# Progressive liver failure induced by everolimus for renal cell carcinoma in a 58-year-old male hepatitis B virus carrier

**DOI:** 10.1007/s12328-013-0371-4

**Published:** 2013-03-14

**Authors:** Shinta Mizuno, Yoshiyuki Yamagishi, Hirotoshi Ebinuma, Nobuhiro Nakamoto, Mai Katahira, Aya Sasaki, Michiie Sakamoto, Hidekazu Suzuki, Takanori Kanai, Toshifumi Hibi

**Affiliations:** 1 Division of Gastroenterology and Hepatology, Department of Internal Medicine, Keio University School of Medicine, Tokyo, 160-8582 Japan; 2 Department of Pathology, Keio University School of Medicine Shinanomachi, Shinjuku-ku, Tokyo, Japan

**Keywords:** Everolimus, Immunosuppressive therapy, Hepatitis B virus, Liver failure, Nucleoside analogue

## Abstract

A 58-year-old man was diagnosed as a hepatitis B virus (HBV) carrier approximately 30 years ago. He was diagnosed with renal cell carcinoma when he was 57 years old. Radical nephrectomy was performed, and everolimus was administered to treat his lung metastasis. After beginning the everolimus, intermittent fever, general fatigue, and jaundice developed. He was admitted under a diagnosis of flare (acute exacerbation) of chronic B hepatitis due to HBV reactivation. Despite intensive care, he died of hepatic failure and fungus infection. The autopsy findings were compatible with hepatic failure due to HBV reactivation by everolimus. Antiviral prophylaxis must be taken into consideration before beginning immunosuppressive therapy such as everolimus in HBV carriers.

## Introduction

Many molecular target agents have been developed for anticancer therapy. Some of them also have immunosuppressive effects. Everolimus is one molecular target drug that inhibits the mammalian target rapamycin (mTOR). It is widely used to treat renal cell carcinoma. In terms of its molecular mechanism, it stabilizes tumor progression, leading to prolonged progression-free survival [1]. The molecule mTOR exists in the middle of the signal cascade following nuclear factor-kappa B (NF-κB). Inhibition of mTOR is also able to block interleukin (IL)-2 signaling, which induces T cell growth and suppresses Th1-cell function [2]. These effects control cellular immunity, and everolimus has both anticancer and immunosuppressive effects.

Reactivation of hepatitis B virus (HBV), which is defined as the recurrence or abrupt rise in HBV replication, occurs both in patients in the inactive carrier state and in those with resolved hepatitis [3]. One million or more HBV carriers are present in Japan. It is thought that many patients do not undergo medical examinations or regular treatment. The Japan de novo Hepatitis B Research Group reported that the prevalence of and mortality associated with fulminant hepatitis were significantly higher among patients with HBV reactivation than among those with acute HBV infection [4]. Furthermore, recent reports showed that patients with malignant lymphoma treated with rituximab, an anti-CD20 agent, had a high risk of HBV reactivation [5, 6]. Reactivation can lead to clinically apparent acute hepatitis, which can be severe and result in acute liver failure and death [3]. Teng et al. [7] reported that inhibition of the mTOR signal could induce HBV replication.

Taken together, mTOR inhibitors, including everolimus, may induce immune suppression in HBV carriers and lead to HBV reactivation. This type of reactivation induced by immunosuppressive therapy in HBV carriers is an important issue that demands prompt action.

## Case report

A 58-year-old Japanese man was diagnosed as hepatitis B surface (HBs) antigen (Ag)–positive by medical examination approximately 30 years ago, but he was not undergoing medical consultation.

He was diagnosed with right renal cell carcinoma at a previous hospital, and radical right nephrectomy was performed when he was 57 years old. Three months after nephrectomy, lung metastases were identified. Interferon (IFN) alpha was started, but it could not control the disease progression. IFN alpha was stopped 2 months later, and sorafenib was started. Eight months later, because of peritoneal dissemination, sorafenib was switched to sunitinib, which was continued for 3 months until general fatigue developed. During the therapies the patient never had liver injury and his serum HBV DNA was not checked.

Approximately 18 months after the diagnosis of renal cell carcinoma, the patient began everolimus therapy. General fatigue and jaundice followed by intermittent fever appeared 5 months after starting everolimus. Laboratory findings showed liver injury, and the patient was admitted to his previous hospital with a diagnosis of acute exacerbation of chronic B hepatitis. Everolimus was stopped and entecavir (1 mg/day, oral administration) was started, but the liver injury and jaundice progressively worsened. Eight days later, he was transferred to our hospital for further medical treatment.

**Fig. 1:**
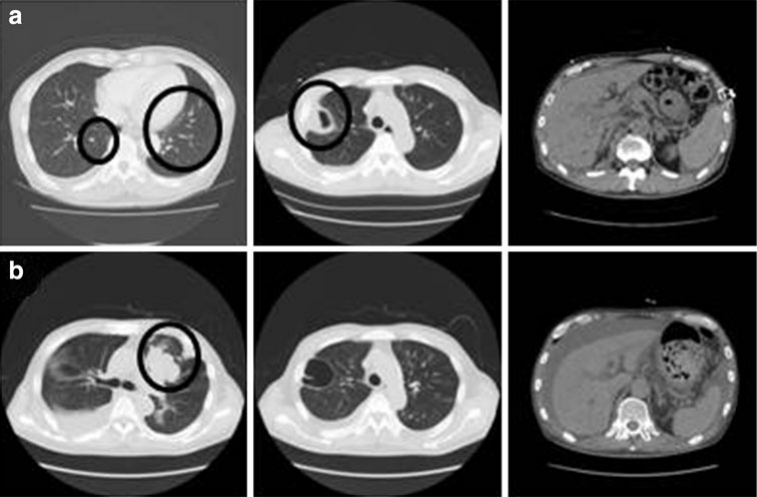
**a** Contrast-enhanced computed tomography (CT) performed on the eighth hospital day showed small nodular lesions scattered in both lung fields (*left*), cavity formation in the upper lobe of the right lung (*middle*), and ascites and slight liver atrophy (*right*). **b** CT performed on the 38th hospital day showed a nodular lesion, lung metastases, and massive pleural effusion in the right thoracic cavity (*left* and *middle*). Liver atrophy was aggravated compared with the above image (*right*) (**b**)

At the time of transfer, his serum aspartate aminotransferase (AST) level was 1920 IU/L, and his alanine aminotransferase (ALT) level was 878 IU/L. He had a marked coagulation disorder with a prothrombin time-international normalized ratio (PT-INR) of 1.83. The serum bilirubin level was elevated with direct bilirubin (D-Bil) predominance; the total bilirubin (T-Bil) was 11.0 mg/dL, and the D-Bil was 7.8 mg/dL. The serum HBV DNA level was 7.2 log copies/mL as measured by real-time polymerase chain reaction, and the HBV genotype was C. Other results of HBV-related serology tests were HBs-Ag-positive, HBe-Ag-negative, HBe-Ab-positive, and HBc-IgM-negative (chemiluminescent enzyme immunoassay). Contrast-enhanced computed tomography (CT) revealed cavity formation in the upper lobe of his right lung, and small nodular lesions were scattered in both lung fields. Ascites and slight liver atrophy were also seen (Fig. 1a).

We continued oral entecavir (0.5 mg/day) and started steroid pulse therapy (methylprednisolone, 1000 mg/day for 3 days with gradual tapering). His serum AST and ALT were gradually decreasing (AST, 101 IU/L; ALT, 112 IU/L) after 2 weeks of therapy, but his serum HBV DNA level was still high (6.7 log copies/mL), and his serum T-Bil was elevated at >20 mg/dL. His PT was still prolonged (PT- % 39). We added IFN beta therapy (3 × 10^6^ IU/day as daily intravenous injections) to decrease the viral load and improve his liver function.

Three weeks after transfer, his plasma (1 → 3)-beta-d-glucan level suddenly increased to 61.2 pg/mL, and his chest X-ray showed a reticular shadow in his left lung. *Aspergillus* infection was suspected because he became positive for serum *Aspergillus* antigen at the same time. We then began amphotericin B at 150 mg/day as a daily intravenous infusion. In addition, he showed a decreased level of consciousness and flapping tremor at the time. That was comparable to hepatic encephalopathy level II(or III).

**Fig. 2:**
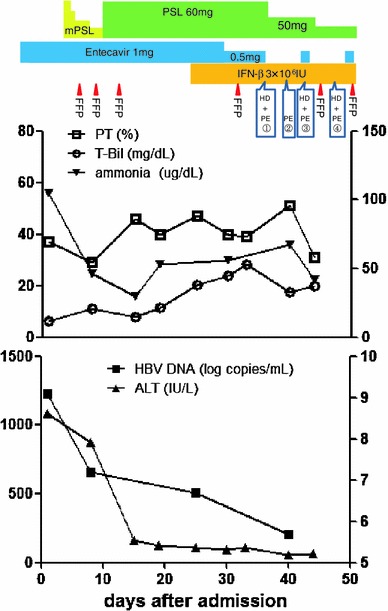
Clinical course of the present case. *Upper panel* shows the treatment course, and *lower panel* shows the course of the laboratory findings. Left longitudinal axis of the *upper line graph* shows PT (%) and T-Bil, and right axis shows ammonia. Left longitudinal axis of the *lower line graph* shows ALT, and right axis shows HBV DNA. *mPSL* methylprednisolone, *PSL* prednisolone, *HD* hemodialysis, *PE* plasma exchange, *FFP* fresh frozen plasma, *T-Bil* total bilirubin, *ALT* alanine aminotransferase, *PT* prothrombin time

Four weeks after transfer, CT revealed that the nodular lesions and lung metastases had extended and that massive pleural effusion had appeared in his right thoracic cavity (Fig. 1b). Furthermore, his renal function was worsening; his serum creatinine level had increased from 1.03 to 3.5 mg/dL. To improve his renal function and support his liver function, we began hemodialysis and plasma exchange. Despite intensive therapy, he died of hepatic failure and fungus infection on the 45th hospital day. Figure 2 shows his clinical course.

**Fig. 3:**
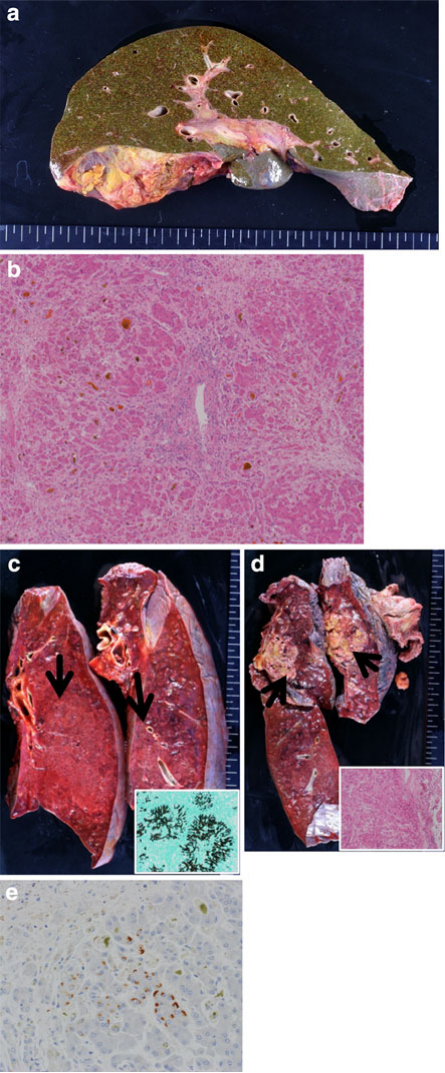
Autopsy findings. **a** Macroscopic view of the liver shows mild atrophy and marked cholestasis. **b** H&E staining of the liver shows massive liver necrosis and moderate lymphocyte infiltration, and cholestasis (magnification 10×). **c** Congested lung and marked overgrowth of *Aspergillus* mycelia in the bilateral lungs. *Arrows* show overgrowth of *Aspergillus* mycelia. Lower right box shows Grocott’s methenamine silver staining of *Aspergillus* mycelia in these lesions. **d** Metastatic lesions in the bilateral lungs. *Arrows* show these lesions. *Lower right box* shows H&E staining of metastatic renal carcinoma in these lesions. **e** HBs-Ag immunostaining in the liver

At autopsy, the liver weight was 1,040 g. The macroscopic view of the liver showed mild liver atrophy and cholestasis (Fig. 3a). The pathological findings showed massive liver necrosis and moderate lymphocyte infiltration and cholestasis (Fig. 3b). There was alveolar hemorrhage, lung congestion, and marked overgrowth of *Aspergillus* mycelia in both lungs. There were large metastatic lesions in the left hilar region, left thoracic wall, and right lung. The pathological findings revealed that the renal cell carcinoma had also spread to left adrenal gland, cardiac muscle of the left ventricle, and bilateral hilar lymph nodes (Fig. 3c, d). Positive HBs-Ag immunostaining for HBs-Ag was seen (spread) in the liver (Fig. 3e).

## Discussion

In the present case, everolimus was started for treatment of metastatic renal cell carcinoma, and HBV reactivation accompanied by severe liver dysfunction subsequently developed. The risk of fulminant hepatitis is significantly higher in HBV reactivation than in acute HBV infection, as described in Introduction [4]. Lamivudine, a nucleoside analogue, may reduce the risk for HBV reactivation of HBs-Ag-positive patients treated with chemotherapy [8, 9]. Lubel et al. [10] stated that prevention of HBV reactivation must be considered during immunosuppressive therapy or chemotherapy. EASL Clinical Practice Guidelines recommend that HBs-Ag-positive candidates for chemotherapy and immunosuppressive therapy should undergo pre-emptive nucleoside analogue administration during therapy and for 12 months after cessation of therapy [11]. Moreover, Li et al. [12] reported that entecavir is more effective than lamivudine in preventing hepatitis B reactivation in patients with lymphoma under chemotherapy. EASL Clinical Practice Guidelines also recommend that patients with a high HBV DNA level and/or repeated cycles of immunosuppression should be protected with a nucleoside analogue with high viral potency and a high barrier resistance; i.e., entecavir or tenofovir. A Japanese study group also recommended pre-emptive nucleoside analogue administration for HBs-Ag-positive patients before receiving immunosuppressive therapy or chemotherapy [13, 14]. The present patient was administered entecavir and IFN after HBV reactivation and severe liver dysfunction developed.

Although anti-HBV therapy and intensive liver and renal support (i.e., plasma exchange and hemodialysis) were performed, the patient developed liver failure and died. Autopsy findings showed massive liver necrosis equivalent to fulminant hepatitis. As mentioned above, everolimus may have a strong potential for immune suppression, and in the present case, drug-induced HBV reactivation with liver failure occurred. It is suggested that antiviral therapy may not be effective once HBV reactivation with liver dysfunction develops. The serum HBV DNA level did not decrease after starting entecavir in the present case. Remarkable necrosis and an inflammatory reaction secondary to *Aspergillus* infection were observed in his lungs; he finally developed respiratory failure in addition to liver failure, leading to his death. No previous history of liver injury before starting everolimus and positive immunostaining of HBs Ag with massive liver necrosis may support the incidence of HBV replication induced by everolimus treatment.

Because Drug Information warns that everolimus can cause hepatitis virus reactivation, there are no previous articles of case reports on everolimus-related HBV reactivation. Taken into consideration of the above-mentioned findings, this patient should have received anti-HBV prophylaxis with a nucleoside analogue such as entecavir or lamivudine before starting everolimus because he was HBs-Ag-positive.

In the clinical trial of everolimus, one HBV carrier died of everolimus-induced HBV reactivation. Detailed information about that case cannot be acquired because it was part of a clinical trial.

This case report is the first to include detailed clinical information. We also obtained the pathological autopsy findings of HBV reactivation by everolimus. This case raises an alert over the importance of prophylactic administration of a nucleoside analogue to HBs-Ag-positive patients. Baseline HBV serology must be tested for all patients who may receive everolimus, and a nucleoside analogue should be started for HBs-Ag-positive patients before treatment to decrease the risk of HBV reactivation.

We need to treat this case as a lesson, and hepatologists must enlightened other doctors about a standard procedure of decreasing that risk. It’s preferable that doctors who consider the possibility of starting immunosuppressive therapy to their patients must check their HBV serology whether their HBs-Ag is positive or negative.
